# Spectrophotometric Determination of Iron(II) and Cobalt(II) by Direct, Derivative, and Simultaneous Methods Using 2-Hydroxy-1-Naphthaldehyde-p-Hydroxybenzoichydrazone

**DOI:** 10.1155/2012/981758

**Published:** 2012-02-27

**Authors:** V. S. Anusuya Devi, V. Krishna Reddy

**Affiliations:** ^1^Department of Chemistry, S.E.A. College of Engineering and Technology, Bangalore 560049, India; ^2^Department of Chemistry, Sri Krishnadevaraya University, Anantapur 515003, India

## Abstract

Optimized and validated spectrophotometric methods have been proposed for the determination of iron and cobalt individually and simultaneously. 2-hydroxy-1-naphthaldehyde-p-hydroxybenzoichydrazone (HNAHBH) reacts with iron(II) and cobalt(II) to form reddish-brown and yellow-coloured [Fe(II)-HNAHBH] and [Co(II)-HNAHBH] complexes, respectively. The maximum absorbance of these complexes was found at 405 nm and 425 nm, respectively. For [Fe(II)-HNAHBH], Beer's law is obeyed over the concentration range of 0.055–1.373 **μ**g mL^−1^ with a detection limit of 0.095 **μ**g mL^−1^ and molar absorptivity **ɛ**, 5.6 × 10^4^ L mol^−1^ cm^−1^. [Co(II)-HNAHBH] complex obeys Beer's law in 0.118–3.534 **μ**g mL^−1^ range with a detection limit of 0.04 **μ**g mL^−1^ and molar absorptivity, **ɛ** of 2.3 × 10^4^ L mol^−1^ cm^−1^. Highly sensitive and selective first-, second- and third-order derivative methods are described for the determination of iron and cobalt. A simultaneous second-order derivative spectrophotometric method is proposed for the determination of these metals. All the proposed methods are successfully employed in the analysis of various biological, water, and alloy samples for the determination of iron and cobalt content.

## 1. Introduction

Iron and cobalt salts are widely used in industrial materials [1, 2], paint products [3], fertilizers, feeds, and disinfectants. They are important building components in biological systems [4]. Special cobalt-chromium-molybdenum alloys are used for prosthetic parts such as hip and knee replacements [5]. Iron-cobalt alloys are used for dental prosthetics [6]. There has been growing concern about the role of iron and cobalt in biochemical and environmental systems. Normally small amounts of iron and cobalt are essential for oxygen transport and enzymatic activation, respectively, in all mammals. But excessive intake of iron causes siderosis and damage to organs [7]. A high dosage of cobalt is very toxic to plants and moderately toxic to mammals when injected intravenously. Hence, quantification of various biological samples for iron and cobalt is very important to know their influence on these systems.

A good number of reviews have been made on the use of large number of chromogenic reagents for the spectrophotometric determination of iron and cobalt. Some of the recently proposed spectrophotometric methods for the determination of iron [8–15] and cobalt [16–22] are less sensitive and less selective. We are now proposing simple, sensitive and selective direct and derivative spectrophotometric methods for the determination of iron(II) and cobalt(II) in various complex materials using 2-hydroxy-1-naphthaldehyde-p-hydroxybenzoichydrazone as chromogenic agent. We are also reporting a highly selective second-order derivative method for the simultaneous determination of iron and cobalt in different samples.

## 2. Experimental

### 2.1. Preparation of Reagents

0.01 M iron(II) and cobalt(II) solutions were prepared by dissolving appropriate amounts of ferrous ammonium sulphate (Sd. Fine) in 2 M sulphuric acid and cobaltous nitrate (Qualigens) in 100 mL distilled water. The stock solutions were diluted appropriately as required. Other metal ion solutions were prepared from their nitrates or chlorides in distilled water. 1% solution of cetyltrimethylammonium bromide (CTAB), a cationic surfactant in distilled water is used. Buffer solutions of pH 1–10 are prepared using appropriate mixtures of 1 M HCl–1 M CH_3_COONa (pH 1–3.0), 0.2 M CH_3_COOH, 0.2 M CH_3_COONa (pH 3.5–7.0), and 1 M NH_4_OH and 1 M NH_4_Cl (pH 7.5–10.0). HNAHBH was prepared by mixing equal amounts of 2-hydroxy-1-naphthaldehyde in methanol and p-hydroxybenzoichydrazide in hot aqueous ethanol in equal amounts and refluxing for three hours on water bath. A reddish brown coloured solid was obtained on cooling. The product was filtered and dried. It was recrystallized from aqueous ethanol in the presence of norit. The product showed melting point 272–274°C.

**Scheme 1:**
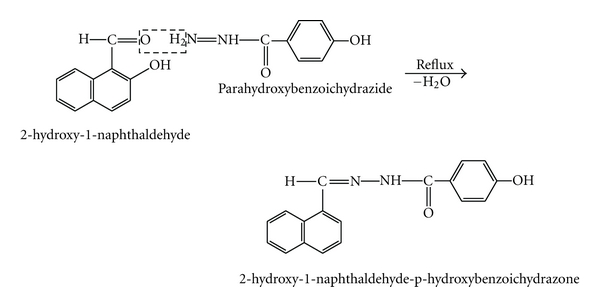


The structure of the synthesized HNAHBH was determined from infrared and NMR spectral analysis. 1 × 10^−2^ M solution of the reagent was prepared by dissolving 0.306 g in 100 mL of dimethylformamide (DMF). Working solutions were prepared by diluting the stock solution with DMF (see Scheme 1).

### 2.2. Preparation of Sample Solutions

#### 2.2.1. Soil Samples

 The soil sample (5.0 g) was weighed into a 250 mL Teflon high-pressure microwave acid digestion bomb and 50 mL aquaregia were added. The bomb was sealed tightly and then positioned in the carousel of a microwave oven. The system was operated at full power for 30 minutes. The digested material was evaporated to incipient dryness. Then, 50 mL of 5% hydrochloric acid was added and heated close to boiling to leach the residue. After cooling, the residue was filtered and washed two times with a small volume of 5% hydrochloric acid. The filtrates were quantitatively collected in a 250 mL volumetric flask and diluted to the mark with distilled water.

#### 2.2.2. Alloy Steel Sample Solution

 A 0.1–0.5 g of the alloy sample was dissolved in a mixture of 2 mL HCl and 10 mL HNO_3_. The resulting solution was evaporated to a small volume. To this, 5 mL of 1 : 1 H_2_O and H_2_SO_4_ mixture was added and evaporated to dryness. The residue was dissolved in 15 mL of distilled water and filtered through Whatman filter paper no. 40. The filtrate was collected in a 100 mL volumetric flask and made upto the mark with distilled water. The solution was further diluted as required.

#### 2.2.3. Food and Biological Samples

 A wet ash method was employed in the preparation of the sample solution. 0.5 g of the sample was dissolved in a 1 : 1 mixture of nitric acid and perchloric acid. The solution was evaporated to dryness, and the residue was ashed at 300°C. The ash was dissolved in 2 mL of 1 M sulphuric acid and made up to the volume in a 25 mL standard flask with distilled water.

#### 2.2.4. Blood and Urine Samples

Blood and urine samples of the normal adult and patient (male) were collected from Government General Hospital, Kurnool, India. 50 mL of sample was taken into 100 mL Kjeldal flask. 5 mL concentrated HNO_3_ was added and gently heated. When the initial brief reaction was over, the solution was removed and cooled. 1 mL con. H_2_SO_4_ and 1 mL of 70% HClO_4_ were added. The solution was again heated to dense white fumes, repeating HNO_3_ addition. The heating was continued for 30 minutes and then cooled. The contents were filtered and neutralized with dil. NH_4_OH in the presence of 1-2 mL of 0.01% tartrate solution. The solution was transferred into a 10 mL volumetric flask and diluted to the volume with distilled water.

#### 2.2.5. Water Samples

Different water samples were collected from different parts of Anantapur district, A. P, India and filtered using Whatman filter paper.

#### 2.2.6. Pharmaceutical Samples

A known quantity of the sample was taken in a beaker and dissolved in minimum volume of alcohol. Then added 3 mL of 0.01 M nitric acid and evaporated to dryness. The dried mass was again dissolved in alcohol. This was filtered through Whatman filter paper, and the filtrate was diluted to 100 mL with distilled water. The lower concentrations were prepared by the appropriate dilution of the stock solution.

### 2.3. Apparatus

 A Perkin Elmer (LAMBDA25) spectrophotometer controlled by a computer and equipped with a 1 cm path length quartz cell was used for UV-Vis spectra acquisition. Spectra were acquired between 350–600 nm (1 nm resolution). ELICO model LI-120 pH-meter furnished with a combined glass electrode was used to measure pH of buffer solutions.

## 3. Results and Discussions

Iron(II) and cobalt(II) react with HNAHBH forming reddish brown and yellow coloured complexes. The colour of the complexes was stable for more than two days.

### 3.1. Direct Method of Determination of Iron(II)

The absorption spectrum of [Fe(II)-HNAHBH] shows maximum absorbance at 405 nm. The preliminary investigations indicate that the absorbance of the complex is maximum and stable in pH range of 4.5–5.5. Hence pH 5.0 was chosen for further studies. A considerable increase in the colour intensity in the presence of 0.1% CTAB was observed. Studies on reagent (HNAHBH) concentration effect revealed that a maximum of 15-fold excess reagent is required to get maximum and stable absorbance for the complex. From the absorption spectra of [Fe(II)-HNAHBH] the molar absorptivity, coefficient *ε* is calculated as 5.6 × 10^4^ L mol^−1^ cm^−1^. Variable amounts of Fe(II) were treated with suitable amounts of reagent, surfactant, and buffer and the validity of Beer's law was tested by plotting the measured absorbance values of the prepared solutions against concentration of Fe(II). The calibration curve was linear over the range 0.055–1.373 *μ*g mL^−1^. The composition of the complex [Fe(II) : HNAHBH] was determined as 2 : 3 by Job's continuous variation method and the stability constant of the complex was calculated as 1.8 × 10^18^. Other analytical results are presented in Table 5.

#### 3.1.1. Effect of Diverse Ions in the Determination of Iron by Direct Method

 Numerous cations and anions were added individually to the experimental solution containing 0.558 *μ*g mL^−1^ of iron and the influence was examined (Table 1). All the anions and many cations were tolerable in more than 100 fold excess. The tolerance limits of some ions were in the range of 5–50 folds. Some of the metal ions, which strongly interfered, could be masked using appropriate masking agents.

#### 3.1.2. Determination of Iron in Surface Soil and Alloy Steels by Direct Spectrophotometric Method

 The applicability of the developed direct method was evaluated by applying the method for the analysis of some surface soil and alloy steel samples for their iron content. Different aliquots of sample solutions containing suitable amounts of iron were treated with known and required volume of HNAHBH at pH 5.0 and 0.1% CTAB and diluted to 10 mL with distilled water. The absorbance of the resultant solutions was measured at 405 nm, and the amount of iron present was computed from the predetermined calibration plot. The results were compared with the certified values and presented in Tables 2 and 3.

### 3.2. Determination of Iron(II) by Derivative Method

**Figure 1:**
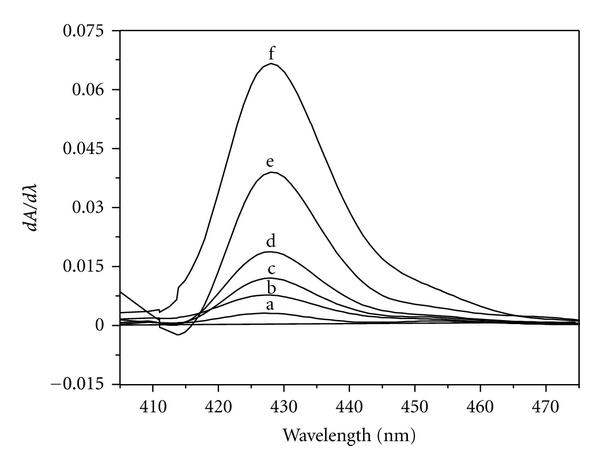
First-order derivative spectra of [Fe(II)-HNAHBH]. Amount of Fe(II) *μ*g mL^−1^: a = 0.027; b = 0.055; c = 0.11; d = 0.22; e = 0.33; f = 0.88.

**Figure 2:**
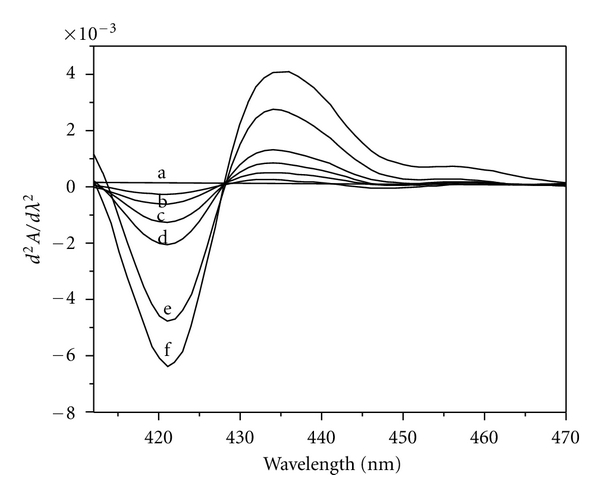
Second-order derivative spectra of [Fe(II)-HNAHBH]. Amount of Fe(II) *μ*g mL^−1^: a = 0.027; b = 0.055; c = 0.11; d = 0.22; e = 0.33; f = 0.88.

**Figure 3:**
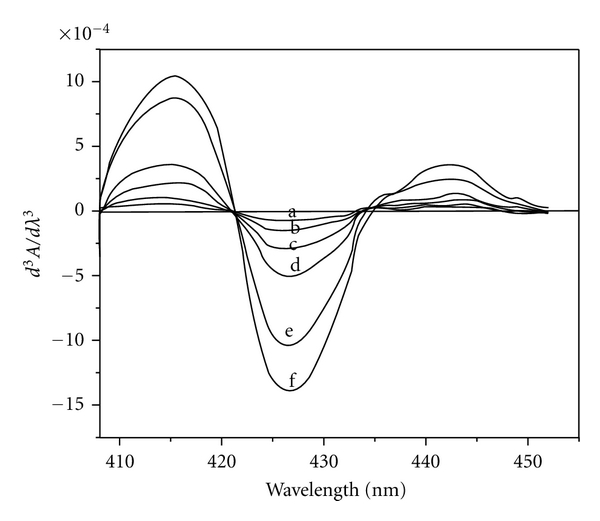
Third-order derivative spectra of [Fe(II)-HNAHBH]. Amount of Fe(II) *μ*g mL-1: a = 0.027; b = 0.055; c = 0.11; d = 0.22; e = 0.33; f = 0.88.

 Different amounts of Fe(II) (0.027–1.375 *μ*g mL^−1^) were treated with suitable amounts of HNAHBH in buffer solutions of pH 5.0 along with 0.1% CTAB and made upto 10 mL with distilled water. 1st, 2nd, and 3rd order derivative spectra were recorded in the wavelength region 350–600 nm. The first-order derivative spectra showed maximum derivative amplitude at 427 nm (Figure 1). The second-order derivative spectra gave one large trough at 421 nm and a large crust at 435 nm with zero cross at 428 nm (Figure 2). A large crust at 415 nm and a large trough at 426 nm with zero cross at 421 nm were observed for the third-derivative spectra (Figure 3). Hence Fe(II) was determined by measuring the derivative amplitudes at 427 nm for 1st order, at 421 nm and 435 nm for 2nd order, and at 415 nm and 426 nm for 3rd order spectra.

#### 3.2.1. Determination of Iron(II)

The derivative amplitudes measured at the analytical wavelengths as mentioned above for different derivative spectra were plotted against the amount of Fe(II). The calibration plots are linear in the range 0.027–1.375 *μ*g mL^−1^. All the derivative methods are found to be more sensitive with a wider Beer's law range than the zero order method (Table 5)

#### 3.2.2. Effect of Foreign Ions in Derivative Method of Determination of Iron

 The influence of some of the cations, which showed serious interference in zero order method, on the derivative amplitudes was studied by the reported methods and the results obtained are shown in Table 4. It can be observed from the table that large number of ions showed significantly high-tolerance limits in some of the derivative methods.

#### 3.2.3. Determination of Iron in Food and Biological Samples by First Order Derivative Method

Known aliquots of the prepared food and biological sample solutions were treated with suitable volumes of HNAHBH, buffer solution, and CTAB surfactant and diluted to the volume in 10 mL volumetric flasks. The first-order derivative spectra were recorded, and the derivative amplitudes were measured at analytical wave lengths. The amounts of Fe(II) in the samples were computed from predetermined calibration plots and presented in Table 6. The food and biological samples were further analyzed by Atomic Absorbance Spectrophotometric method, and the results obtained were compared with those of the present method. 

### 3.3. Direct Method of Determination of Cobalt(II)

 [Co(II)-HNAHBH] complex shows maximum absorbance at 425 nm. Maximum and stable absorbance of the complex is achieved in the pH range of 5.0–7.0. Hence pH 6.0 was chosen for further studies. A marginal increase in the absorbance was observed in presence of 0.15% of CTAB. 10-folds excess of HNAHBH is sufficient to get maximum absorbance. Molar absorptivity of the complex was calculated as 2.3 × 10^4^ L mol^−1^ cm^−1^. Beer's law is tested taking the different amounts of Co(II) in presence of suitable buffer, surfactant, and HNAHBH, linearity of the calibration curve is found between 0.118–3.534 *μ*g mL^−1^ with a detection limit of 0.04 *μ*g mL^−1^ and determination limit 0.124 *μ*g mL^−1^ (Table 11), which shows the sensitivity of the present method. The stoichiometry of the complex was found to be 2 : 3 (Metal : Ligand) by Job's method. The stability constant is calculated as 7.7 × 10^19^. 

#### 3.3.1. Effect of Foreign Ions in the Determination of Cobalt by Direct Method

 The effect of various anions and cations normally associated with Co(II) on the absorbance of the experimental solution was studied. The tolerance limits of the tested foreign ions, which bring about a change in the absorbance by ±2% were calculated and presented in Table 7. 

 Among anions, except EDTA and citrate, all other tested ions were tolerable in more than 200-fold excess. EDTA and citrate were tolerable in 144- and 150-fold excess, respectively. Of the tested cations, some of them did not interfere even when present in more than 500 fold excess, many cations were tolerable between 10–80-folds. Cations which interfere seriously are masked with suitable anions. 

#### 3.3.2. Determination of Cobalt in Surface Soil, Blood and Urine Samples by Direct Method

Suitable aliquots of the soil, blood, and urine sample solutions were taken and analyzed for cobalt content by the proposed method, and the results are presented in Tables 8 and 9. The soil solutions were further analyzed by a reference method [23], and biological samples were analyzed by flame atomic absorption spectrophotometer, and the results obtained were compared with those of present method, which indicate the acceptability of the present method. 

### 3.4. Determination of Cobalt by Derivative Method

**Figure 4:**
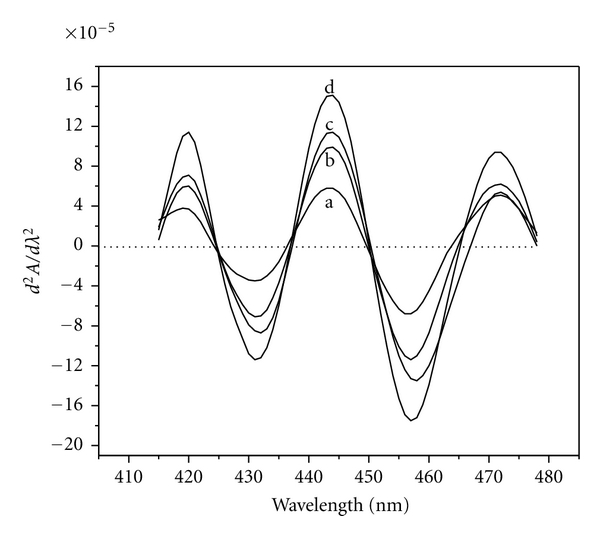
Second-order derivative spectra of [Co(II)-HNAHBH]. Amount of Co(II) *μ*g mL^−1^: a = 0.059, b = 0.118, c = 0.236, and d = 0.354.

**Figure 5:**
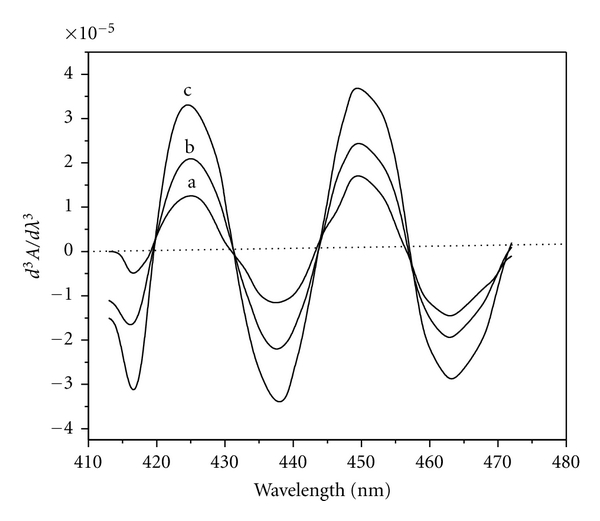
Third-order derivative spectra of [Co(II)-HNAHBH]. Amount of Co(II) *μ*g mL^−1^: a = 0.059, b = 0.118, c = 0.236, and d = 0.354.

 Variable amounts (0.059–4.712 *μ*g mL^−1^) of Co(II), taken in different 10 mL volumetric flasks, were treated with optimal amounts of reagent HNAHBH at pH 6.0 in presence of 0.15% CTAB, and the derivative spectra were recorded in the wavelength region 350–600 nm against reagent blank. The second-derivative curves (Figure 4) gave a trough at 431 nm and a crust at 443 nm with a zero cross at 437 nm. In the third-derivative spectra (Figure 5), maximum amplitude was observed at 424 nm, 437 nm, 449 nm, and at 462 nm with zero crossings at 431 nm, 443 nm, and 456 nm. 

#### 3.4.1. Determination of Cobalt

 The derivative amplitudes measured for different concentrations of Co(II) at appropriate wavelengths for 2nd and 3rd order derivative spectra were plotted against the amount of Co(II) which gave linear plots in the specified concentration regions. All the parameters like detection limit, correlation coefficient, and relative standard deviation values are presented in Table 11. 

#### 3.4.2. Effect of Foreign Ions

The selectivity of the derivative methods was evaluated by studying the effect of metal ions closely associated with cobalt on its derivative amplitudes under experimental conditions. The results are presented in Table 10. The results show that the tolerance limits of Th(IV), U(VI), Mn(II), Cu(II), Ni(II), Zn(II), Sn(II), In(II), Ga(III) and V(V) which interfere seriously in zero order method were greatly enhanced in the derivative methods indicating the greater selectivity of derivative methods over the direct method. 

#### 3.4.3. Determination of Cobalt in Water and Pharmaceutical Samples by Second-Order Derivative Method

Suitable aliquots of water and pharmaceutical samples were taken and analysed for cobalt by second-order derivative method. The results obtained in the analysis of water samples by the proposed method are presented in Table 12 and the validity of the results was evaluated by adding known amounts of Co(II) and calculating their recovery percentage. The results obtained with pharmaceutical samples were compared with those obtained by AAS method and presented in Table 13. 

### 3.5. Simultaneous Second-Order Derivative Spectrophotometric Determination of Iron(II) and Cobalt(II)

Iron and cobalt occur together in many real samples like alloy steels, biological fluids, and environmental samples. In most cases, the characterizations of these samples include the determination of their metal ion content. The need for the determination of iron and cobalt in environmental and biochemical materials has increased after reports on different roles of these metals in human health and diseases. We are now reporting a simple, sensitive, and selective second-order derivative spectrophotometric method for the simultaneous determination of Fe(II) and Co(II) using HNAHBH without the need to solve the simultaneous equations. 

#### 3.5.1. Derivative Spectra 

**Figure 6:**
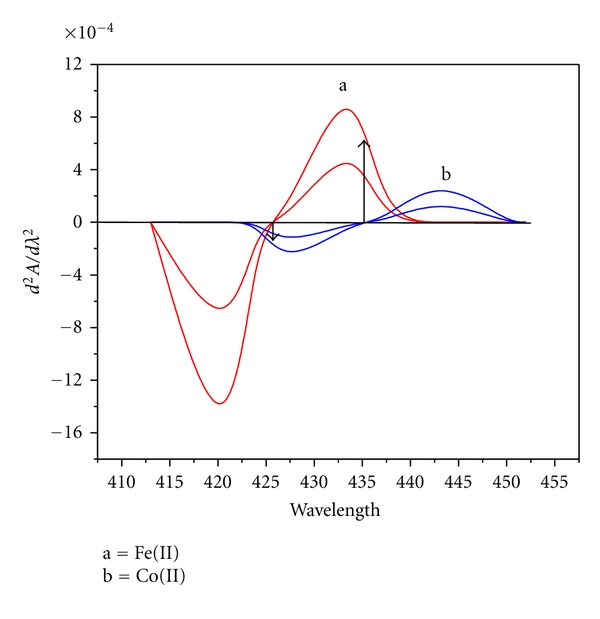
Second-order derivative spectra of (a) [Fe(II)-HNAHBH] and (b) [Co(II)-NAHBH]. Amount of Fe(II) (*μ*g mL^−1^): 0.055, 0.11; Co(II) (*μ*g mL^−1^): 3.53; 4.719.

The 2nd order derivative spectra recorded for [Fe(II)-HNAHBH] and [Co(II)-HNAHBH] at pH 5.5 showed sufficiently large derivative amplitude for cobalt at 426 nm while the Fe(II) species exhibit zero amplitude (Figure 6). At 436 nm, maximum derivative amplitude was noticed for Fe(II) where there was no amplitude for Co(II). This facilitates the determination of Fe(II) and Co(II) simultaneously by measuring the second-derivative amplitudes of binary mixtures containing Fe(II) and Co(II)at 436 nm and 426 nm, respectively. 

#### 3.5.2. Determination of Fe(II) and Co(II)

Aliquots of solutions containing 0.055–1.650 *μ*g mL^−1^ of Fe(II) or 0.117–4.719 *μ*g mL^−1^ of Co(II) were transferred into a series of 10 mL calibrated volumetric flasks. HNAHBH (1 × 10^−2^ M, 0.3 mL), CTAB (1%, 1.5 mL), and buffer solution (pH 5.5, 4 mL) were added to each of these flasks and diluted to the mark with distilled water. The zero-crossing points of [Fe(II)-HNAHBH] and [Co(II)-HNAHBH] species were determined by recording the second-order derivative spectra of both the systems with reference to the reagent blank. Calibration plots for the determination of Fe(II) and Co(II) were constructed by measuring the second-derivative amplitudes at zero crossing points of [Co(II)-HNAHBH] (436 nm) and [Fe(II)-HNAHBH] (426 nm), respectively, and plotting against the respective analyte concentrations. Fe(II) and Co(II) obeyed Beer's law in the range 0.055–1.650 *μ*g mL^−1^ and 0.117–4.719 *μ*g mL^−1^ at 436 nm and 426 nm, respectively. Calibration plots were constructed for the standard solutions containing Fe(II) alone and in the presence of 0.589 *μ*g mL^−1^ of Co(II). Similarly, the calibration graphs were constructed for standards containing Co(II) alone and in the presence of 0.330 *μ*g mL^−1^ of Fe(II). The slopes, intercepts, and correlation coefficients of the prepared calibration plots were calculated and given in Table 14. The derivative amplitudes measured at 436 nm and 426 nm were found to be independent of the concentration of Co(II) and Fe(II), respectively. This allows the determination of Fe(II) and Co(II) in their mixtures without any significant error and without the need for their prior separation.

#### 3.5.3. Simultaneous Determination of Co(II) and Fe(II) in Binary Mixtures

 Fe(II) and Co(II) were mixed in different proportions and then treated with required amount of HNAHBH in the presence of buffer solution (pH 5.5) and 0.15% of CTAB and diluted to the volume in 10 mL volumetric flasks. The second-order derivative spectra for these solutions were recorded (350–600 nm) and the derivative amplitudes were measured at 436 nm and 426 nm. The amounts of Fe(II) and Co(II) in the mixtures taken were calculated from the measured derivative amplitudes using the respective predetermined calibration plots. The results obtained along with the recovery percentage and relative errors are presented in Table 15, which indicate the usefulness of the proposed method for the simultaneous determination of Fe(II) and Co(II) in admixtures. 

#### 3.5.4. Simultaneous Determination of Iron and Cobalt in Alloy Samples

The developed second-order derivative spectrophotometric method was employed for the simultaneous determination of iron and cobalt in some alloy samples. Appropriate volumes of the alloy samples were treated with required amount of HNAHBH at pH 5.5 in the presence of 0.15% CTAB and diluted to 10 mL in standard flasks. The second-derivative curves for the resultant solutions were recorded, and the derivative amplitudes were measured at 426 nm and 436 nm. The amounts of iron and cobalt in the samples were evaluated with the help of predetermined calibration plots and presented in Table 16.

## 4. Conclusions

 A comparison of the analytical results of the proposed methods was made with those of some of the recently reported spectrophotometric methods and presented in Table 17. The data in the above table reveals that the proposed method of determination of iron is more sensitive than those reported by Malik and Rao [27], Patil and Dhuley [28], Nagabhushana et al. [29], Wang et al. [30], Zhang et al. [31], and Martins et al. [36]. The methods proposed by Katmal and Hoyakava [24], Morales and Toral [25], and Reddy et al. [26] are more sensitive than the present method. However they are less selective than the proposed method as they suffer interference from W(VI), Pd(II), Cr(III), Tl(I), Pb(II), Bi(III), Hg(II), Mo(VI), EDTA, CN^−^. Regarding the determination of cobalt, the present method is more sensitive than those reported by Malik et al [16], Patil and Sawant [32], Adinarayana Reddy et al. [33], and Prabhulkar et al. [22]. However, the preset method is less sensitive than the methods reported by Guzor and Jin [21] and Qiufen et al. [34], but these methods are less selective due to the interference of many cations and anions. The results obtained in the simultaneous determination of Fe(II) and Co(II) are well comparable with the reported methods. Above all most of the reported methods involve extraction into spurious organic solvents where as the present methods are simple, nonextractive, and reasonably accurate. 

## Figures and Tables

**Table 1:** Tolerance limits of foreign ions, Amount of Fe(II) taken = 0.558 *μ*g mL^−1^ pH = 5.0.

Foreign ion	Tolerance limit (*μ*g mL^−1^)	Foreign ion	Tolerance limit (*μ*g mL^−1^)	Foreign ion	Tolerance limit (*μ*g mL^−1^)
Sulphate	1440	Na(I)	1565	La(III)	18
Iodide	1303	Mg(II)	1460	Ag(I)	15
Phosphate	1424	Ca(II)	1440	Hg(II)	16
Thiosulphate	1424	K(I)	1300	U(VI)	6,60^a^
Tartrate	1414	Ba(II)	1260	Mn(II)	4,55^a^
Thiourea	1140	Pd(II)	63	Th(IV)	3,50^a^
Bromide	1138	Cd(II)	45	In(III)	4,60^a^
Nitrate	930	Bi(III)	42	Sn(II)	<1,50^a^
Carbonate	900	W(VI)	37	Co(II)	<1,55^a^
Thiocyanate	870	Hf(IV)	36	Ni(II)	<1,60^b^
Chloride	531	Ce(IV)	28	Zn(II)	<1,80^b^
Fluoride	285	Cr(VI)	27	Al(III)	<1,45^a^
EDTA	124	Mo(VI)	22	Cu(II)	<1,50^a^
Citrate	115	Zr(IV)	19		
Oxalate	95	Sr(II)	18		

In the presence of a = 500 *μ*g of tartrate, b = 400 *μ*g of thiocyanate.

**Table 2:** Determination of iron in surface soil.

Sample	Source of the sample	Amount of iron (mg Kg^−1^) ± SD*
S1	Groundnut cultivation soil Akuthotapalli, Anantapur	40.98 ± 0.45
S2	Cotton cultivation soil, Singanamala, Anantapur district,	27.48 ± 0.36
S3	Sweet lemon cultivation soil, Garladinne, Anantapur distrcct	44.88 ± 0.24
S4	Paddycultivation soil Garladinne, Anantapur district	20.86 ± 0.37

*Average of five determinations.

**Table 3:** Determination of iron in alloy steels.

Alloy steel composition (%)	Amount of iron (%)		
	Certified value	Present method ± SD*	Relative error (%)
*High tensile steel * BY0110-1 (42.98 Zn, 19.89 Si, 0.351 Pb, 0.06 Sn, 0.04 Cd, 0.024 As, 0.14 Cu, and 4.13 Fe) YSBC19716	4.13	4.06 ± 0.021	0.17
(34.26 Zn, 0.38 Si, 1.2 Cd, 48.57 Sb, 0.95 S, and 0.32 F) GSBD33001-94	34.26	4.18 ± 0.022	0.01
(9.29 Al, 1.04 Ca, 9.53 Fe)	9.53	9.46 ± 0.039	0.08

*Average of five determinations.

**Table 4:** Tolerance limits of some cations in derivative methods.

Foreign ion	Tolerance limit (in folds)			
	Direct method	First derivative	Second derivative	Third derivative
Ag(I)	14	18	35	22
Hg(II)	11	20	40	30
U(VI)	11	12	25	18
Mn(II)	7	20	16	20
Th(IV)	5	10	16	20
In(III)	7	28	48	34
Au(III)	4	35	55	28
Sn(II)	<1	8	15	22
Co(II)	<1	interfere	7	15
Ni(II)	<1	interfere	5	10
Cu(II)	<1	5	12	10

**Table 5:** Analytical characteristics of [Fe(II)-HNAHBH].

Parameter	Direct method	First derivative	Second derivative		Third derivative	
	405 nm	427 nm	421 nm	435 nm	415 nm	426 nm
Beer's law range (*μ*g mL^−1^)	0.055–1.373	0.027–1.376	0.027–1.376	0.027–1.376	0.027–1.376	0.027–1.376
Molar absorptivity, (L mol^−1^ cm^−1^)	5.6 × 10^4^			—	—	—
Sandell's sensitivity, (*μ*g cm^−2^)	0.0012			—	—	—
Angular coefficient (m)	0.974	0.072	0.006	0.093	0.002	0.085
Y-intercept (b)	0.0047	−0.0045	−0.1 × 10^−3^	−0.1 × 10^−3^	0.2 × 10^−4^	0.9 × 10^−3^
Correlation coefficient	0.9997	0.9999	0.9999	0.9999	0.9999	0.9999
RSD (%)	2.19	0.85	0.76	0.89	1.31	1
Detection limit (*μ*g mL^−1^)	0.065	0.1	0.022	0.0268	0.036	0.304
Determination limit, (*μ*g mL^−1^)	0.197	0.3	0.068	0.8	0.11	0.914
Composition (M : L)	2 : 3	—	—		—	
Stability constant	1.8 × 10^18^	—	—		—	

**Table 6:** Determination of iron in food and biological samples.

Samples	Amount of iron(*μ*g mL^−1^) ± SD (*n* = 4)					
	Found			Recovered		
	present	AAS	Added	present	AAS	% recovery
Wheat	6.68 ± 0.18	6.40 ± 0.09	5	11.40 ± 1.15	11.28 ± 0.10	97.6
Rice	14.10 ± 40.25	16.46 ± 0.18	5	19.7 ± 40.27	21.04 ± 0.48	102
Tomato	11.96 ± 1.20	12.68 ± 0.14	5	17.68 ± 0.25	17.44 ± 0.95	104
Orange	18.12 ± 0.73	16.94 ± 0.66	5	22.20 ± 0.75	22.26 ± 0.68	96
Banana	10.12 ± 1.46	11.4 ± 0.12	5	14.86 ± 1.45	15.86 ± 1.46	98.3
						
Prostate gland	3.26 ± 0.28	2.98 ± 0.08	6.5	10.04 ± 1.68	9.54 ± 0.94	103
Benign (enlarged prostate gland	12.38 ± 3.18	13.15 ± 1.18	6.5	17.96 ± 1.56	20.18 ± 1.66	95.12

**Table 7:** Tolerance limits of foreign ions, amount of Co(II) taken = 1.767 *μ*g mL^−1^, pH = 6.0.

Foreign ion	Tolerance limit (*μ*g mL^−1^)	Foreign ion	Tole limit (*μ*g mL^−1^)	Foreign ion	Toler limit (*μ*g mL^−1^)
Tartrate	1707	Na(I)	1666	Au(III)	20
Phosphate	1425	Mg(II)	1530	Sr(II)	18
Sulphate	1440	Ca(II)	1426	Mo(VI)	15
Oxalate	1320	K(I)	1200	Tl(IV)	13
Bromide	1198	Ba(II)	1162	Pd(II)	11,100^c^
Thiourea	1140	Hf(IV)	72	Th(IV)	6,60^a^
Thiosulphate	1120	Se(IV)	64	U(VI)	5,60^a^
Nitrate	930	Cd(II)	56	Mn(II)	5,50^a^
Chloride	525	W(VI)	55	Cu(II)	2,50^a^
Carbonate	300	Zr(IV)	46	Ni(II)	<1,80^b^
Fluoride	285	Pb(II)	42	Zn(II)	<1
EDTA	144	Hg(II)	40	Sn(II)	<1
Citrate	115	Cr(VI)	26	In(III)	<1,60^a^
		Bi(III)	21	Ga(III)	<1,50^a^
		Ru(III)	21	V(V)	<1,50^b^

In the presence of *a* = 700 *μ*g of tartrate, *b* = 400 *μ*g of oxalate and *c* = 500 *μ*g of thiourea.

**Table 8:** Determination of cobalt in surface soil samples.

Sample and source		Cobalt (*μ*g mL^−1^)	
		Present method*	Reference method [23]
S1	Agricultural land (red soil Anantapur.)	16.48 ± 0.030	17.20 ± 0.024
S2	Agricultural land (black soil, Tadipatri.)	24.15 ± 0.026	23.68 ± 0.022
S3	Riverbed soil (Tungabhadra river, Kurnool)	14.68 ± 0.034	15.26 ± 0.018
S4	Industrial soil (electroplating industry, Anantapur)	118.40 ± 0.042	122.12 ± 0.029

*Average of four determinations.

**Table 9:** Analysis of blood and urine samples for cobalt content.

Sample source	Sample	Cobalt (*μ*g mL^−1^)	
		Present method ± SD (*n* = 5)	AAS method ± SD (*n* = 5)
Normal adult (male)	Blood	2.44 ± 0.020	2.48 ± 0.014
	Urine	0.38 ± 0.010	0.35 ± 0.022
Anemia patient (female)	Blood	0.86 ± 0.020	0.92 ± 0.020
	Urine	0.24 ± 0.030	0.23 ± 0.014
Paralysis patient	Blood	8.46 ± 0.030	8.65 ± 0.032
	Urine	2.65 ± 0.020	2.43 ± 0.025
Pulmonary patient	Blood	4.32 ± 0.015	4.26 ± 0.010
	Urine	1.96 ± 0.022	2.04 ± 0.018

**Table 10:** Tolerance limit of foreign ions (*μ*g mL^−1^).

Diverse ion	Zero order	Second derivative	Third derivative
Th(IV)	6	55	35
U(VI)	5	40	45
Mn(II)	5	60	20
Cu(II)	2	80	45
Ni(II)	<1	30	50
Zn(II)	<1	45	20
Sn(II)	<1	25	18
In(III)	<1	15	28
Ga(III)	<1	20	35
V(V)	<1	15	20

**Table 11:** Analytical characteristics of [Co(II)-HNAHBH].

Parameter	Direct method	Second derivative		Third derivative	
	425 nm	431 nm	443 nm	437 nm	449 nm
Beer's law range (*μ*g mL^−1^)	0.118–3.534	0.059–4.712	0.059–4.712	0.059–1.380	0.056–1.380
Molar absorptivity, (L mol^−1^ cm^−1^)	2.3 × 10^4^	—	—	—	—
Sandell's sensitivity, *μ*g cm^−2^	0.003	—	—	—	—
Angular coefficient (m)	0.375	0.0003	0.093	0.0002	0.009
Y-intercept (b)	0.0197	3.2 × 10^−5^	−0.9 × 10^−4^	−0.2 × 10^−4^	−0.9 × 10^−4^
Correlation coefficient	0.9999	0.999	0.9999	0.9999	0.9999
RSD (%)	1.37	1.84	4.3	1.15	7.6
Detection limit (*μ*g mL^−1^)	0.04	0.06	0.13	0.04	0.21
Determination limit, (*μ*g mL^−1^)	0.124	0.18	0.39	0.114	0.65
Composition (M : L)	2 : 3	—	—		—
Stability constant	7.7 × 10^19^	—	—		—

**Table 12:** Determination of cobalt in environmental water samples.

Sample	cobalt (*μ*g mL^−1^)			
	Added	Found	Recovery (%)	RSD (%)
Tap water (municipality water supply, Anantapur)	0.0	0.32	—	2.5
	1.5	1.80	98.90	1.8
	3.0	3.35	100.90	3.0
	4.5	4.83	100.20	2.2

River water (Penna, Tadipatri.)	0.0	1.52	—	3.0
	1.5	3.00	99.34	1.6
	3.0	4.55	100.66	2.8
	4.5	5.95	98.84	4.0

Drain water (vanaspati industry, Tadipatri.	0.0	3.60	—	1.7
	1.5	5.31	104.12	3.2
	3.0	6.48	98.18	2.5
	4.5	8.07	99.63	3.6

**Table 13:** Determination of cobalt in pharmaceutical tablets.

Sample (mg/tablet)	Amount of cobalt (*μ*g mL^−1^)		
	Reported	Found*	Relative error (%)
Neurobion forte (cyanocobalamine-15 mg)	7.45	7.4	−0.67
Basiton forte (cyanocobalamine-15 mg)	7.42	7.24	−2.42

*Average of four determinations.

**Table 14:** Linear regression analysis of the determination of Fe(II) and Co(II) in mixture by second derivative spectrophotometry.

Metal ion determined	Wave length (nm)	Other metal present (*μ*g mL^−1^)		Slope	Intercept	Correlation coefficient
		Fe(II)	Co(II)			
Fe(II)	436			3.9 × 10^−3^	2.4 × 10^−4^	0.9994
			0.589	3.2 × 10^−3^	1.9 × 10^−4^	0.9995
Co(II)	426			1.4 × 10^−4^	2.3 × 10^−6^	0.9999
		0.33		1.4 × 10^−4^	2.0 × 10^−6^	0.9998

**Table 15:** Simultaneous second-order derivative spectrophotometric determination of Fe(II) and Co(II).

Amount taken (*μ*g mL^−1^)		Amount found* (*μ*g mL^−1^)		Relative error (%)	
Fe(II)	Co(II)	Fe(II)	Co(II)	Fe(II)	Co(II)
0.06	0.59	0.053 (96.3)	0.572 (98.8)	−3.6	−2.8
0.12	0.59	0.120 (103.4)	0.592 (100.5)	3.44	0.5
0.23	0.59	0.230 (99.1)	0.586 (99.4)	−0.86	−0.5
0.33	0.59	0.334 (101.2)	0.572 (98.8)	1.21	−2.8
0.44	0.59	0.441 (100.2)	0.590 (100.1)	0.22	0.2
0.55	0.59	0.542 (98.5)	0.586 (99.3)	−1.45	−0.5
0.33	0.59	0.328 (99.3)	1.120 (94.9)	−0.60	−0.7
0.33	1.18	0.326 (89.6)	2.280 (96.6)	−1.21	−5.0
0.33	2.36	0.324 (98.1)	3.600 (101.7)	−1.81	−3.3
0.33	3.54	0.336 (101.8)	4.670 (98.9)	1.81	1.6
0.33	4.72	0.332 (100.6)	4.670 (98.9)	0.60	−1.0

*Average of four determinations.

**Table 16:** Determination of iron and cobalt in alloy samples.

Sample (composition)	Amount (%)				Relative error (%)	
	Certified		Found (*n* = 3) ± SD			
	Fe(II)	Co(II)	Fe(II)	Co(II)	Fe(II)	Co(II)
Elgiloy-M (20 Cr; 15 Ni; 0.15 C; 2 Mn; 7 Mo;.05 Be)	15	40	14.82 ± 0.15	39.39 ± 0.20	1.33	1.52
Rim alloy (17 Mo; 3Mn)	68	12	69.28 ± 0.86	12.08 ± 0.38	1.88	0.66
Sofcomag 25 (Fe and Co)	75	25	73.89 ± 1.38	25.98 ± 0.86	1.48	3.92
Sofcomag 49 (Fe and Co)	51	49	52.12 ± 0.35	49.18 ± 0.06	2.18	0.36

**Table 17:** Comparision of the results with already reported methods.

Metal ion	Reagent	*λ*_max⁡_ (nm)	pH/medium	Aqueous/extraction	Beer's law *μ*g mL^−1^	*ε* × 10^4^L mol^−1^ cm^−1^	Interference	Reference
Fe(II)	Thiocyanate-phenanthroline	520	—	Aqueous	0–24	1.87	—	[10]
Fe(II)	2-[2-(3,5-Dibromopyridyl-azo]-5-dimethyl amino-benzoic acid	615	2.0–7.0	Extraction	0–5.5	9.36	Tl(I), Zn(II), Cr(III), W(VI), Co(II), Cu(II), Ni(II), and Pd(II)	[24]
Fe(II)	1,10-Phenanthroline and picrate	510	2.0–9.0	Extraction	0.1–3.6	13	EDTA, CN^−^	[25]
Fe(II)	4-(2-Pyridylazo)resorcinol	505	6.0–7.5	Extraction	0–2.0	6	Ni(II), Co(II), Pb(II), and EDTA	[26]
Fe(II)	1,10-Phenanthroline-tetraphenylborate	515	4.25	Aqueous	2.24–37.29	1.2	—	[27]
Fe(II)	1,3-Diphenyl-4-carboethoxy pyrazole-5-one	525	3.5–4.0	Aqueous	0.5–10	1.156	Cu(II), Co(II), Zn(II), Mo(VI), EDTA	[28]
Fe(II)	Dyformyl hydrazine	470	7.3–9.3	Aqueous	0.25–13	0.3258	—	[29]
Fe(II)	4,7-Diphenyl-1,10-phenanthroline and tetraphenylborate	534	—	Extraction	0–20.0	2	—	[30]
Fe(II)	Thiocyanate-acetone	480	HClO_4_	Aqueous	—	2.1	Cu(II), NO_2_^−^, S_2_O_3_^−2^, H_2_PO_4_^−2^, and C_2_O_4_^−2^	[31]
Fe(II)	2-Hydroxy-1-naphthaldehyde-p-hydroxybenzoic hydrazone	405	5	Aqueous	0.05–1.37	5.6	Sn(II), Co(II) Ni(II) Zn(II) Al(III) Cu(II)	Present method
Co(II)	Sodium isoamyl xanthate	400	4.5–9.0	Aqueous	3.0–35	1.92		[16]
Co(II)	2-Pyridine carboxalde hydeisonicotinyl-hydrazine	346	9	Aqueous	0.01–2.7	7.1	Au(III), Ag(I), Pt(III)	[18]
Co(II)	2-Hydroxy-1-naphthalidene salicyloyl hydrazone	430	8.0–9.0	Extraction	0–10	0.16		[22]
Co(II)	Pyridine-2-acetaldehyde salicyloyl hydrazone	415	1.0–6.0	Extraction	0.5–7.0	1.04	—	[32]
Co(II)	Bis-4-phenyl-3-thiosemicarbazone	400	4	—	0.6–6.0	2.2	—	[33]
Co(II)	2-Hydroxy-l-naphthalidene-salicyloylhydrazone	430	8.0–9.0	Extraction	0–10	1.6 × 10^3^	—	[22]
Co(II)	2-(2-Quinolynylazo)-5-dimethylamino aniline	625	5.5	Extraction	0.01–0.6	4.3	Many cations and anions	[34]
Co(II)	2-Hydroxy-3-methoxy benzaldehyde thiosemicarbazone	390	6	Aqueous	0.06–2.35	2.74	—	[35]
Co(II)	2-Hydroxy-1-naphthaldehyde-p-hydroxybenzoic hydrazone	425	5	Aqueous	0.12–3.54	2.3	Ni(II), Zn(II), Sn(II), In(III), and Ga(IIII)	Present method
